# Pre-Pandemic Distribution of Bacterial Species in Nasopharyngeal Swab Specimens from Pediatric and Adult Patients Detected via RT-PCR Using the Allplex Respiratory Panel

**DOI:** 10.3390/life13091840

**Published:** 2023-08-30

**Authors:** Dong-Hyun Lee, Young-Jin Choi, Jieun Kim, Eunhee Han, Mi-Hyun Bae

**Affiliations:** 1 Department of Laboratory Medicine, Gyeongsang National University Hospital, Gyeongsang National University School of Medicine, Jinju 52727, Republic of Korea; 2 Department of Pediatrics, Hanyang University Guri Hospital, Hanyang University College of Medicine, Guri 11923, Republic of Korea; 3 Division of Infectious Disease, Department of Internal Medicine, Hanyang University Guri Hospital, Hanyang University College of Medicine, Guri 11923, Republic of Korea; 4 Department of Laboratory Medicine, Daejeon St. Mary’s Hospital, College of Medicine, The Catholic University of Korea, Seoul 06591, Republic of Korea; 5 Department of Laboratory Medicine, Hanyang University Guri Hospital, Hanyang University College of Medicine, Guri 11923, Republic of Korea

**Keywords:** respiratory infection, bacteria, nasopharyngeal swab, RT-PCR, panel-based assay, Allplex respiratory panel

## Abstract

**Background:** Recently, panel-based molecular diagnostics for the simultaneous detection of respiratory viruses and bacteria in nasopharyngeal swab (NPS) specimens have been highlighted. We identified the distribution of bacterial species in NPS specimens collected from pediatric and adult patients by employing RT-PCR (Allplex respiratory panel 4, RP4, Seegene) to estimate its applicability in a panel-based assay for detecting respiratory viruses. **Methods:** We used 271 and 173 NPS specimens from pediatric and adult patients, respectively. The results of the Allplex RP4 panel using NPS (NPS-RP4) from adult patients were compared with those of the Seeplex PneumoBacter ACE Detection assay (Seegene), which used sputum for testing (sputum-Seeplex). **Results:** A total of 147 specimens (54.2%) were positive for the NPS-RP4 panel in pediatric patients. There were 94, 77, 10, 3, 3, and 2 specimens that were positive for *Haemophilus influenzae* (HI), *Streptococcus pneumoniae* (SP), *Mycoplasma pneumoniae* (MP), *Chlamydia pneumoniae* (CP), *Bordetella pertussis* (BP), and *B. parapertussis* (BPP), respectively. Among 173 adult patients, 39 specimens (22.5%) were positive in the NPS-RP4. Thirty specimens were positive for HI, and 13 were positive for SP. One specimen tested positive for both MP and *Legionella pneumophila* (LP). CP, BP, and BPP results were all negative. However, 126 specimens (72.8%) had positive results with sputum-Seeplex (99 SP, 59 HI, three LP, and two MP), and the overall percentage of agreement between the two assays was 39.3% in the adult patients. **Conclusions:** Bacterial species in NPS from more than half of pediatric patients were detected. Performing the Allplex RP4 assay with NPS revealed additional respiratory bacteria that are not detected in current clinical practices, which do not include bacterial testing, demanding the use of sputum specimens. However, the use of NPS showed low agreement with standard assays using sputum in adult patients. Thus, more research is needed to develop a reliable RT-PCR method using NPS specimens in adult patients.

## 1. Introduction

Even before the coronavirus 2019 (COVID-19) pandemic, respiratory infections were a primary cause of death [1,2]. Respiratory infections can be caused by various pathogens, including bacteria and viruses, or by a combination of the two [3,4], and the causal organism cannot be distinguished by clinical symptoms and signs alone [5,6]. Accurate and fast detection of the etiological agent is critical for timely patient management, secondary infection dissemination prevention, and reduction of hospital stay [7,8,9].

Conventional techniques, such as culture-based isolation and antigen-based testing, are routine tests for patients with suspected respiratory infections. Culture methods can detect various bacteria in comparison to molecular methods, such as real-time polymerase chain reaction (RT-PCR), which can only detect specific organisms. However, these conventional tests are time-consuming and labor-intensive, and their sensitivities and specificities are not uniform [10]. Molecular assays for multiplex detection of respiratory pathogens have been widely used in Korea for more than a decade [11]. The Allplex Respiratory Panel (Seegene, Seoul, Republic of Korea) is a typical RT-PCR-based multiplex assay that has been recently used in Korea [12]. Three respiratory virus panels (RP1, RP2, and RP3) of the Allplex assay cover 16 respiratory viruses and three influenza virus A subtypes, with samples collected using a nasopharyngeal swab (NPS). This kit was introduced in Korea in 2017 and has since then been widely used. However, the respiratory panel for the detection of bacteria was approved later than the virus panels in Korea. Allplex respiratory panel 4 (RP4), a CE-certified assay covering seven respiratory bacteria, was launched overseas earlier than in Korea and is used for clinical practice in more than 70 countries in 2022 [13]. In 2019, RP4 was approved for clinical use in Korea and named Allplex™ PneumoBacter Assay with minor modifications for *Streptococcus pneumoniae* and *Bordetella pertussis* and to exclude *B. parapertussis*. As these Allplex respiratory panels use the same PCR conditions, they can be applied in panel-based assays using a single specimen.

Over the past decade, panel-based molecular assays for the simultaneous detection of respiratory viruses and bacteria have received widespread attention. The FilmArray Respiratory Panel (BioFire Diagnostics, Salt Lake City, UT, USA) is the market leader in panel-based assays, which were released in Korea in 2013. This panel-based assay simultaneously detects 17 viruses and three bacteria that cause atypical pneumonia from one NPS specimen in one step within 1 h [14]. However, the high cost of reagents is the main obstacle to its establishment as a routine test in Korea.

Bacterial respiratory infections have a poorer prognosis than viral infections and can be fatal if diagnosis and treatment are delayed; therefore, in Korea, empirical antibiotics are commonly prescribed for patients with respiratory symptoms without diagnostic testing for causative pathogens. Furthermore, conventional standard specimens for viral and bacterial assays differ; NPSs are used for viral assays, whereas sputum specimens are used for bacterial assays, which are difficult to obtain from pediatric patients. As a result, diagnostic tests for respiratory bacteria are not performed in pediatric patients, or invasive procedures are performed to obtain nasopharyngeal aspirates. Thus, information on bacterial distribution in pediatric patients with respiratory infections in Korea is scarce. The use of empirical antibiotics without identifying etiologic pathogens also increases the rate of antibiotic resistance. For example, *Mycoplasma pneumoniae* strains from Korea showed high macrolide resistance rates [15]. However, the introduction of panel-based assays for the detection of respiratory viruses and bacteria using NPSs allows rapid diagnosis in patients with respiratory symptoms, even in pediatric patients, and could contribute to alleviating antibiotic overuse.

In the past decade, pathogens, such as the measles virus and poliovirus, which were thought to be nearly eradicated, have reemerged, and some of them triggered worldwide epidemics [16,17]. In accordance with this trend, after many years of not being reported in Korea, *B. pertussis* has recently been detected in adult patients [18]. Although *B. pertussis* is potentially more serious in pediatric patients, it is not properly managed in Korea due to the common omission of assays that detect respiratory bacteria, unlike those used for the detection of respiratory viruses. 

Thus, we aimed to identify the distribution of bacterial species in NPS from pediatric and adult patients with respiratory infections using the Allplex RP4 assay. 

## 2. Materials and Methods

### 2.1. Patients and Specimens

This study used NPS samples from patients who were admitted to Hanyang University Guri Hospital with acute respiratory symptoms and signs between August and November 2018, including fever, cough, sputum, dyspnea, and rhinorrhea. NPS specimens were obtained by trained medical staff according to the guideline, as “gently insert the swab along the nasal septum, just above the floor of the nasal passage, to the nasopharynx, until resistance is felt. Insert the swab into the nostril, parallel to the palate” [19]. The swabs were transferred using an eSwab 482CE preservation medium (Copan, Brescia, Italy). After routine RT-PCR for respiratory virus (RP1, RP2, and RP3), the samples were kept at −70 °C and then subsequently subjected to RP4 testing for analysis. The effect of freezing storage on the specimens was examined by generating respiratory virus assay results for specimens both before freezing and after thawing. Specimens were collected on the day of hospitalization prior to the administration of antibiotics. The history of antibiotic use before hospitalization was collected via interview or from medical records obtained from primary clinics. Clinical data pertaining to diagnosis, chest radiograph results, serological results of *M. pneumoniae* IgM, and antibiotic use were obtained from electronic medical records. The results of the Seeplex PneumoBacter ACE Detection assay using sputum specimens were obtained from electronic medical records of adult patients to compare with those of the RP4 assay using NPS. This study was approved by the Institutional Review Board (IRB) of Hanyang University Guri Hospital (IRB No. 2018-06-031). The requirement for informed consent was waived by the IRB.

### 2.2. Allplex RP4 Assay Using NPS

The STARMag Universal Cartridge Kit (Seegene) was employed to extract nucleic acids using an automated Microlab STARlet IVD (Seegene) that can process 30 samples per run. A total of 200 μL of each sample was extracted and eluted with 100 μL of elution buffer, as recommended by the manufacturer [13]. RT-PCR was performed using a CFX96 System (Bio-Rad, Hercules, CA, USA). A single reaction RP4 can detect and differentiate seven different bacteria: *S. pneumoniae*, *M. pneumoniae*, *Legionella pneumophila*, *Haemophilus influenzae*, *Chlamydophila pneumoniae*, *B. pertussis*, and *B. parapertussis*. A volume of 8 μL of the extracted DNA/RNA in a 25 μL final volume was used for the reaction. The results were automatically evaluated using Seegene software (Seegene Viewer V2.0), and the entire process took 210 min. Samples with a cycle threshold (Ct) ≤ 42 were considered positive, while samples without a Ct or with a Ct > 42 were considered negative.

### 2.3. Seeplex PneumoBacter ACE Detection Assay Using Sputum

Respiratory bacteria were detected in sputum from adult patients at a reference laboratory (Seegene Medical Foundation, Seoul, Republic of Korea). The Seeplex PneumoBacter ACE Detection assay is a multiplex PCR approach that detects six bacteria, excluding *B. parapertussis*, as visualized by electrophoresis. The assay was performed according to the manufacturer’s instructions [20]. PCRs using a 20-μL volume containing 3 μL DNA and 17 μL PCR premix were performed under the following conditions: 94 °C for 15 min, 40 cycles of three steps (94 °C for 30 s, 60 °C for 1 min 30 s, and 72 °C for 1 min 30 s), and a final extension at 72 °C for 10 min. PCR products were observed using agarose gel electrophoresis on ethidium bromide-stained 2% agarose gels. The sizes of amplicons were the following: *M. pneumoniae*, 583 bp; *L. pneumophila*, 472 bp; *S. pneumoniae*, 350 bp; *H. influenzae*, 257 bp; *B. pertussis*, 200 bp; and *C. pneumoniae*, 146 bp. Each amplification reaction mixture contained internal control DNA, and each run was accompanied by positive and negative controls.

### 2.4. Statistical Analysis

Statistical analyses were performed using the Statistical Package for the Social Sciences (version 18.0; Chicago, IL, USA). Categorical variables were summarized as absolute counts and percentages, whereas continuous variables were presented as means with ranges. The overall percentage agreement was calculated using a cross-table analysis. Pearson’s chi-squared test was used to analyze categorical data that contained 5 or more values in 80% of the table cells but no cells with zero expected count. All tests were two-sided, and a *p* < 0.05 was considered statistically significant.

## 3. Results

### 3.1. Pediatric Patients

A total of 271 pediatric patients were enrolled consecutively in this study. Among them, the mean age was 3.4 years (range: 0–17), and 117 patients (43.2%) were male, and 154 patients (56.8%) were female (Table 1). Abnormalities were observed in the chest radiographs of sixty patients (22.1%), while 146 patients (53.9%) presented a fever ≥38.6 °C and 38 patients (14.0%) tested positive in the serum *M. pneumoniae* IgM assay. Antibiotics were administered to the majority of patients (n = 238, 87.8%). Cephalosporin was the most commonly prescribed drug (n = 200, 84.0%), followed by macrolide (n = 144, 60.5%) and penicillin series (n = 35, 14.7%). A total of 131 patients (48.3%) were administered a cephalosporin + macrolide regimen on the day of admission. Prevalent diagnoses were bronchitis (n = 52, 19.2%), acute pharyngotonsillitis (n = 48, 17.7%), pneumonia (n = 40, 14.8%), acute gastroenteritis (n = 27, 10.0%), other upper respiratory tract infections (n = 18, 6.6%), and bronchiolitis (n = 13, 4.8%). The ‘other diagnosis’ category includes less common diagnoses, such as urinary tract infection (n = 9, 3.3%), herpangina (n = 8, 3.0%), meningitis (n = 7, 2.6%), sinusitis (n = 6, 2,2%), exanthem subitum (n = 5, 1.8%), acute otitis media (n = 3, 1.1%), and sepsis (n = 3, 1.1%).

Among the pediatric patients, 147 patients (54.2%) obtained a positive result in the NPS-RP4 assay (Table 2). *H. influenzae*, *S. pneumoniae*, *M. pneumoniae*, *C. pneumoniae*, *B. pertussis*, and *B. parapertussis* were found in 94, 77, 10, 3, 3, and 2 patients, respectively (34.7%, 28.4%, 3.7%, 1.1%, 1.1%, and 0.7%, respectively). None of the patients tested positive for *L. pneumophila*. Among the 94 *H. influenzae*-positive and 77 *S. pneumoniae*-positive patients, *H. influenzae* and *S. pneumoniae* were co-detected in 33 patients (12.2%). Among the ten *M. pneumoniae*-positive patients, four showed co-detections: two cases with *H. influenzae* and two with *S. pneumoniae.* All three *C. pneumoniae*-positive patients showed co-detection with *S. pneumoniae*. Among the patients who tested positive for *Bordetella* species, one with *B. pertussis* also tested positive for *H. influenzae*, and one with *B. parapertussis* also tested positive for *S. pneumoniae*.

**Figure 1:**
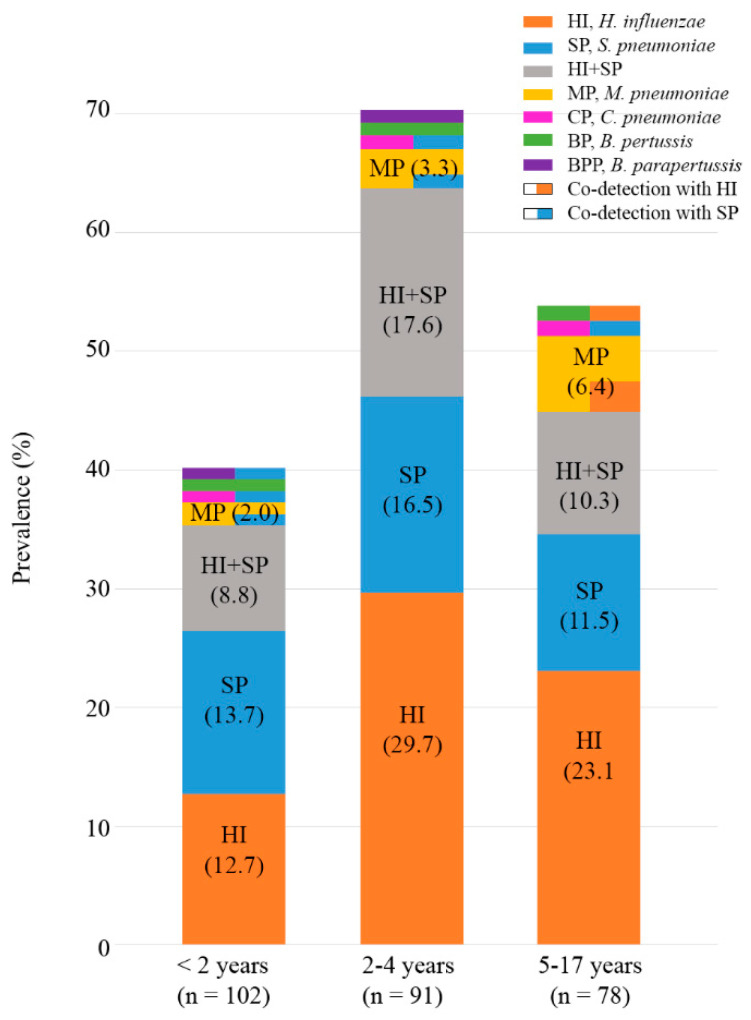
Prevalence of bacterial species in the different age groups of pediatric patients. Overall prevalence was the highest in the 2–4-year age group (70.3%). The most prevalent species in the <2-year age group was *S. pneumoniae* (25.5%) and *H. influenzae* in the 2–4-year (47.3%) and 5–17-year age groups (37.2%). The prevalence of *M. pneumoniae* was the highest in the 5–17-year age group (6.4%).

The prevalence of bacterial species was analyzed according to the age groups of pediatric patients, and the results are described in Figure 1. The overall positive rate was the highest in the 2–4-year age group (70.3%), compared to those in the other age groups (40.2% for <2 years and 53.8% for the 5–17-year age group). In the <2-year age group, the most prevalent species was *S. pneumoniae*, which was detected in 25.5% of patients, including 12 co-detection cases. *H. influenzae* was the most prevalent species in the age groups of 2–4 and 5–17 years. Patients in the 2–4-year age group showed a prevalence of 47.3% for *S. pneumoniae* (27 single detections and 16 co-detections). Patients in the 5–17-year age group showed a prevalence of 37.2% for *S. pneumoniae* (18 single detections and 11 co-detections). The prevalence of *M. pneumoniae* was the highest in the 5–17-year age group (6.4%). The cases of *C. pneumoniae* and *B. pertussis* were equally distributed among the three age groups, with one case in each group. Among the two cases of *B. parapertussis*, one was detected in the <2-year age group and the other was detected in the 2–4-year age group; *B. parapertussis* was not detected in the 5–17-year age group.

Clinical and laboratory findings of the bacterial species detected using the Allplex RP4 assay are described in Table 3. The proportion of male patients was the highest in those positive for *C. pneumoniae* and *B. parapertussis* (100% male for both) and lowest in those positive for *B. pertussis* (33.3% male). The ratio of abnormalities in chest radiographs was higher in patients positive for *M. pneumoniae* (90.0%) compared with those positive for *H. influenzae*, *S. pneumoniae*, and HI + SP (19.0%, 15.8%, and 27.3%, respectively), which was statistically significant according to Pearson’s chi-squared test (*p* < 0.001). The proportion of patients with fever ≥38.6 °C was lowest in patients who tested positive for *S. pneumoniae* (44.7%). Antibiotics were administered to all patients (100%) who tested positive for pathogens that cause atypical pneumonia. The proportion of upper respiratory tract infections, including acute pharyngotonsillitis, other upper respiratory tract infections, sinusitis, and acute otitis media, was the highest in patients positive for *C. pneumoniae* and *B. pertussis* (66.7% for both). The proportion of lower respiratory tract infection, including bronchitis, pneumonia, and bronchiolitis, was higher in patients positive for *M. pneumoniae* (90.0%) compared with those that were positive for *H. influenzae*, *S. pneumoniae*, and HI + SP (39.7%, 36.8%, and 45.5%, respectively), which was statistically significant according to Pearson’s chi-squared test (*p* = 0.020).

### 3.2. Adult Patients

A total of 173 specimens from 173 patients were used in this study. Males outnumbered females by 1.7 times, with 110 (63.6%) males and 63 (36.4%) females, and the mean age was 68.9 (range: 23–92). RP4 assay using NPS (NPS-RP4) revealed positive results for 45 targeted organisms from 39 patients (22.5%), while the Seeplex assay with sputum samples showed positive results for 163 targeted organisms from 126 patients (72.8%) (Table 4). The overall percentage of agreement between the two assays was 39.3%. When considering the Seeplex assay using sputum as the reference method, NPS-RP4 showed a sensitivity of 42.4% (95% confidence interval (CI): 29.6–55.9%) and specificity of 95.6% (95% CI: 90.1–98.6%) to detect *H. influenzae* (Table 4). For *S. pneumoniae*, NPS-RP4 showed a sensitivity of 12.1% (95% CI: 6.4–20.2%) and a specificity of 98.7% (95% CI: 92.7–100%). The sensitivity of NPS-RP4 was 50.0% (95% CI: 1.3–98.7%) and 33.3% (95% CI: 0.8–90.6%) for *M. pneumoniae* and *L. pneumophila*, respectively. The specificities of both the assays for *M. pneumoniae* and *L. pneumophila* were 100%.

## 4. Discussion

The distribution of bacterial species in NPS from pediatric and adult patients with respiratory infections was identified via RT-PCR using the Allplex RP4 assay. Bacterial species were detected in more than half of pediatric patients. *H. influenzae* was the most common bacterial species in both pediatric and adult patients; however, the overall positivity rate in adult patients was notably lower (22.5%) in the NPS-RP4 assay compared to that in the sputum-Seeplex assay (72.8%).

Respiratory infection due to bacteria is less prevalent in children than respiratory viral infection, but it has a poorer prognosis, and progress can be fatal if diagnosis and treatment are delayed [6,21]. Molecular assays of respiratory viruses and bacteria can provide a rapid diagnosis for patients with respiratory symptoms. However, unlike virus assays that use NPS specimens, the standard specimen for bacterial assays is sputum, which is difficult for children to spit out. As a result, many tests for respiratory bacteria are not performed in pediatric patients, or invasive procedure is performed to obtain nasopharyngeal aspirates. Consequently, many pediatric patients receive prophylactic antibiotics without being tested for bacteria, resulting in antibiotic overuse. In this particular clinical situation of pediatric patients, panel-based assays, such as those for respiratory viruses and bacteria, can be valuable diagnostic tools. Because Allplex RP4 successfully detected bacterial pathogens in the NPS of pediatric patients in the current study, it can be used as a panel-based assay in conjunction with Allplex respiratory panels for viruses.

A few studies conducted in Europe [13,17] used NPS to evaluate Allplex RP4. In 156 pediatric patients, a previous study reported that *S. pneumoniae* had the highest detection rate (53.8%), followed by *H. influenzae* (41%) and *H. influenzae* and *S. pneumoniae* co-detection (26.2%) [22]. In the current investigation, the results showed a similar trend in bacterial pathogen distribution in pediatric patients, with the following distribution: *H. influenzae* (34.7%), *S. pneumoniae* (28.4%), and co-detection with *H. influenzae* and *S. pneumoniae* (12.2%). *H. influenzae* and *S. pneumoniae* are two of the most prevalent pathogens responsible for causing invasive infections, including pneumonia [23]. They showed a decreasing trend after the vaccine was introduced, although the prevalence of multidrug-resistant *S. pneumoniae* has steadily increased in Korea [24]. Because these bacteria are commonly found in the nasopharynx, definitive diagnosis using a single molecular assay is difficult. However, this assay may be useful in identifying patients when clinical suspicion exists, and it may also help focus on specific treatment strategies in test-negative patients while reducing empirical antibiotic overuse.

*M. pneumoniae* respiratory infections are typically benign, self-limiting diseases that can be effectively treated with macrolides [25]. However, in some pediatric cases, Mycoplasma infection can progress to a severe, life-threatening infection in patients who are resistant to conventional macrolide treatment [26]. The most common method for detecting *M. pneumoniae* infection is the serological test; however, confirmation relies on diagnostic criteria based on the variation in antibody levels (with a 4-fold increase in the recovery phase) [27]. Therefore, it cannot be guaranteed whether the positive or negative result at the time of detection is a true positive or negative [28]. Thus, a 4-fold increase in antibody levels was confirmed by repeated blood testing during the recovery phase to verify the diagnosis. Of the 10 patients who tested positive for *M. pneumoniae* using RP4, only four tested positive for *M. pneumoniae* IgM upon admission. However, the majority of patients who tested positive for *M. pneumoniae* using the Allplex RP4 assay had lower respiratory tract infections with abnormal chest radiographs in this study. These findings might reflect the clinical significance of *M. pneumoniae* detection in NPSs using molecular assays. In addition, further validation of diagnostic accuracy using a larger patient cohort could change the diagnostic paradigm of *M. pneumoniae*, which could reduce the challenges associated with obtaining two blood tests to check for antibody differences in pediatric patients. 

In Korea, *M. pneumoniae* is one of the most common pathogens that cause community-acquired pneumonia requiring hospitalization in pediatric patients, with large outbreaks in 2011 and 2015 [29]. Furthermore, the recent widespread and frequent use of macrolides has substantially increased the number of resistant strains [28]. Macrolide antibiotics are often prescribed based on the assumption that antibody levels increase after a few days, regardless of the negative results of the initial blood test. Consequently, the number of macrolide-resistant Mycoplasma strains has recently increased. Because doxycycline or quinolone-based drugs cannot be used against resistant strains, pediatric patients may eventually require steroids [26,28]. As a result, the establishment of Allplex RP4 is expected to reduce antibiotic use and the risk of developing antibiotic-resistant strains provided that the infection can be diagnosed in the early stages of the disease. 

*B. pertussis*, a class 2 notifiable infectious disease in Korea, was found in three cases in this investigation. *B. pertussis* is a highly infectious pathogen that causes respiratory diseases and several complications in pediatric patients. The occurrence of the disease was significantly reduced by the introduction of the vaccine in Korea. However, a repeating outbreak pattern has recently been discovered, and an appropriate testing method has not yet been confirmed. A previous study reported that NPS performed similarly in RT-PCR assays targeting the insertion sequence 481 when compared to nasopharyngeal aspirates or induced sputum in infant patients [30]. Thus, in pediatric patients who cannot provide sputum samples, the NPS-based assay, including Allplex RP4, is expected to be a reliable test for detecting pneumonia caused by agents, such as *B. pertussis,* allowing rapid diagnosis and transmission prevention.

The epidemiology of infectious diseases changed globally during and after the COVID-19 pandemic. Viral and bacterial pathogens causing respiratory infections were substantially decreased in the 2020–2021 seasons, but a rebound of various pathogens occurred after the social restrictions were lifted [31,32]. Respiratory viruses, such as the parainfluenza virus, respiratory syncytial virus, and influenza virus, resurfaced during the unforeseen season, with a higher epidemic peak than before the COVID-19 pandemic [33,34,35,36]. As this trend deviates from the established epidemiological seasonality of common respiratory pathogens, clinical diagnoses based on traditional epidemiological data will be more constrained in the post-COVID-19 era. Thus, rapid identification of multiple causative pathogens, including viruses and bacteria, in patients with respiratory symptoms would aid in the timely diagnosis and treatment of patients, as well as effective infection control.

A previous study examined the sensitivity of NPS and sputum samples from adult patients for the detection of *M. pneumoniae*, *L. pneumophila*, and *C. pneumoniae* using the Seeplex PCR assay and determined that sputum was more sensitive than NPS [37]. Another study examined the two specimen types in RT-PCR targeting the autolysin gene of *S. pneumoniae* in elderly patients with community-acquired pneumonia and found that NPS had a lower detection rate and less of the pneumococcal genome than sputum [38]. These previous results complement our findings that the bacterial detection rate with NPS was inferior to that with sputum in adult patients. Because this appears to be caused by the intrinsically smaller quantity of the bacterial genome in NPS than in the sputum of adult patients, further improvements in molecular technology are needed to unify specimens and establish panel-based testing that simultaneously covers pathogens causing upper and lower respiratory tract infections.

This study has several limitations that must be considered. First, there was no comparison with sputum or nasopharyngeal aspirates from pediatric patients. Second, in adult patients, a consistent PCR assay was not performed to compare NPS and sputum. Third, other molecular approaches were not used to confirm the species and quantity of bacterial genome in the NPS. Fourth, comparisons with gold-standard methods, such as culture isolation or urinary antigen tests, were not included in this study. Fifth, this study used NPS specimens that were stored at −70 °C for up to 8 months. The Allplex panels detecting respiratory viruses were used to monitor the effect of freezing, which was performed before freezing and after thawing. Although the results showed minimal discrepancy (0.33%, 28 target discordance over 8436 targets tested) in tests using virus panels, the results obtained with RP4 assay using thawed specimens may have been influenced by the freezing process.

In conclusion, bacterial species were detected in more than half of the NPS specimens collected from pediatric patients. Performing the Allplex RP4 assay with NPS revealed additional respiratory bacteria that are not detected in current clinical practices, which do not include bacterial testing, demanding the collection of sputum specimens. Simultaneous testing using the Allplex virus panels could also aid in diagnosis and reduce the time and cost, as well as the antibiotic overuse. However, the use of NPS showed low agreement with standard assays that use sputum collected from adult patients. Thus, more research is needed to develop a reliable RT-PCR method that uses NPS specimens in adult patients.

## Figures and Tables

**Table 1:** Clinical and laboratory characteristics of pediatric patients.

Characteristics	No. of Patients (%)
Total patients	271
Mean age, years (range)	3.4 (0–17)
Sex	
Male	117 (43.2)
Female	154 (56.8)
Abnormal chest radiograph	60 (22.1)
Fever ≥ 38.6 °C	146 (53.9)
Positive *M. pneumoniae* IgM	38 (14.0)
Antibiotic use	238 (87.8)
Cephalosporin	200 (84.0)
Macrolide	144 (60.5)
Penicillin series	35 (14.7)
Diagnosis	
Bronchitis	52 (19.2)
Acute pharyngotonsillitis	48 (17.7)
Pneumonia	40 (14.8)
Acute gastroenteritis	27 (10.0)
Other upper respiratory infections	18 (6.6)
Bronchiolitis	13 (4.8)
Other *	73 (27.0)

* Other diagnosis includes urinary tract infection, herpangina, meningitis, sinusitis, exanthem subitum, otitis media, and sepsis.

**Table 2:** Results of the Allplex RP4 assay using nasopharyngeal swabs from 271 pediatric patients.

Species	No. of Patients (%)		No. of Co-Detection Cases (%)			
			*H. influenzae*		*S. pneumoniae*	
Negative	124	(45.8)				
Positive	147	(54.2)				
*H. influenzae*	94	(34.7)	33 (12.2)			
*S. pneumoniae*	77	(28.4)				
*M. pneumoniae*	10	(3.7)	2	(0.7)	2	(0.7)
*C. pneumoniae*	3	(1.1)			3	(1.1)
*B. pertussis*	3	(1.1)	1	(0.4)		
*B. parapertussis*	2	(0.7)			1	(0.4)
*L. pneumophila*	0	(0.0)				

**Table 3:** Clinical and laboratory characteristics according to the bacterial species detected in the nasopharyngeal swabs of pediatric patients using the Allplex RP4 assay.

Characteristics	No. of Patients (%)						
	*H. influenzae*	*S. pneumoniae*	HI + SP	*M. pneumoniae*	*C. pneumoniae*	*B. pertussis*	*B. parapertussis*
Sex							
Male	33 (56.9)	22 (57.9)	20 (60.6)	5 (50.0)	3 (100)	1 (33.3)	2 (100)
Female	25 (43.1)	16 (42.1)	13 (39.4)	5 (50.0)	-	2 (66.7)	-
Abnormal chest radiograph	11 (19.0)	6 (15.8)	9 (27.3)	9 (90.0) *	-	-	1 (50.0)
Fever ≥ 38.6 °C	31 (53.4)	17 (44.7)	19 (57.6)	6 (60.0)	2 (66.7)	-	1 (50.0)
Positive *M. pneumoniae* IgM	6 (10.3)	8 (21.1)	7 (21.2)	4 (40.0)	1 (33.3)	-	-
Antibiotic use	49 (84.5)	33 (86.8)	30 (90.9)	10 (100)	3 (100)	3 (100)	2 (100)
Diagnosis							
Upper respiratory tract infection	20 (34.5)	9 (23.7)	10 (30.3)	-	2 (66.7)	2 (66.7)	1 (50.0)
Lower respiratory tract infection	23 (39.7)	14 (36.8)	15 (45.5)	90 (90.0) *	1 (33.3)	1 (33.3)	-
Other	15 (25.9)	15 (39.5)	8 (24.2)	1 (10.0)	-	-	1 (50.0)
Total No. of patients	58 (100)	38 (100)	33 (100)	10 (100)	3 (100)	3 (100)	2 (100)

HI, *H. influenzae*; SP, *S. pneumoniae*. * p < 0.05 using Pearson’s chi-squared test.

**Table 4:** Comparison of the results of the Allplex RP4 assay using nasopharyngeal swabs from 173 adult patients based on the Seeplex assays using sputum samples as the reference method.

NPS-RP4		Sputum-Seeplex		Sensitivity, % (95% CI)	Specificity, % (95% CI)
		Positive	Negative		
*H. influenzae*	Positive	25	5	42.4 (29.6–55.9)	95.6 (90.1–98.6)
	Negative	34	109		
*S. pneumoniae*	Positive	12	1	12.1 (6.4–20.2)	98.7 (92.7–100)
	Negative	87	73		
*M. pneumoniae*	Positive	1	0	50.0 (1.3–98.7)	100 (97.9–100)
	Negative	1	171		
*L. pneumophila*	Positive	1	0	33.3 (0.8–90.6)	100 (97.9–100)
	Negative	2	170		

NPS, Nasopharyngeal swab; CI, confidence interval.

## Data Availability

Data associated with this manuscript can be obtained from the corresponding author upon reasonable request.
